# Role of Pacific SSTs in improving reconstructed streamflow over the coterminous US

**DOI:** 10.1038/s41598-018-23294-6

**Published:** 2018-03-21

**Authors:** Sudarshana Mukhopadhyay, Jason M. Patskoski, A. Sankarasubramanian

**Affiliations:** Department of Civil, Construction and Environmental Engineering North Carolina State University, Raleigh, NC 27695–7908 USA

## Abstract

Reconstructed annual streamflows using tree-ring chronologies provide useful information on moisture availability during the pre-historic period, but they have limitations in estimating high flows due to the upper bound on soil water holding capacity and trees’ metabolic growth limits. We propose a hybrid approach that uses tree-ring chronologies and climatic indices for improving high flows in 301 basins whose annual streamflows are modulated by ENSO and/or PDO. The hybrid decomposition approach relies on separating the moisture supply into the basin as outside-the-region moisture and within-the-region moisture with the former being estimated by SST indices and the latter being estimated by tree-ring chronologies. Analyses over the 301 stations from coterminous US show that the proposed approach improves the high flows and improves the overall error in the reconstructed streamflows. Potential utility of the improved reconstructed annual streamflows with improved high flows is also discussed.

## Introduction

**Figure 1:**
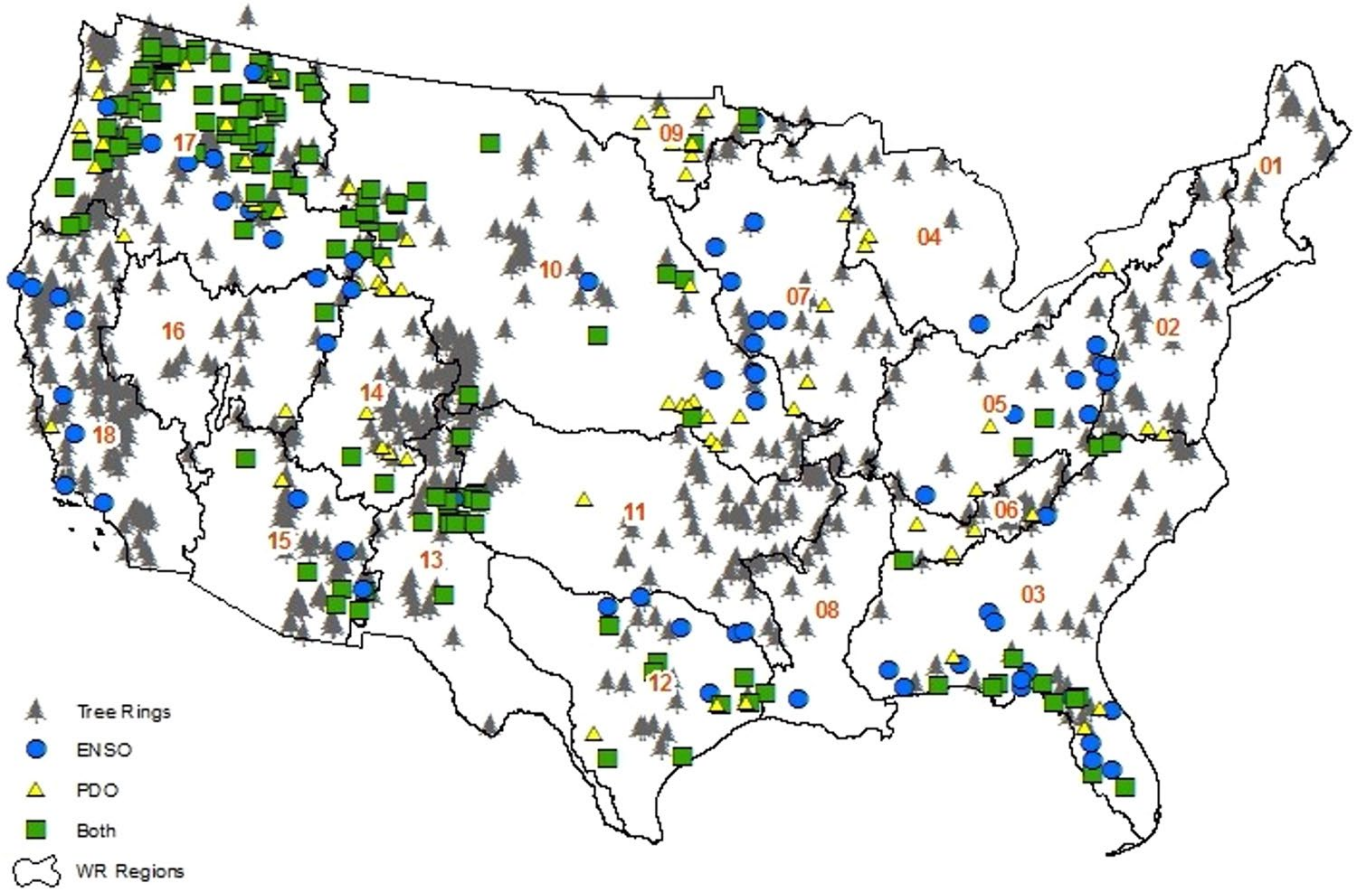
Locations of HCDN stations and tree-ring chronology sites across 18 water resources regions (orange) used in this study with blue circles (yellow triangles) [green squares] indicating the basins significantly influenced by ENSO (PDO) [ENSO and PDO]. This map is created using software suite ArcGIS 10.2.2 for Desktop, version number 10.2.2.3552 (http://www.esri.com/en/arcgis/products/arcgis-pro/overview).

Annual streamflow records provide crucial information on the available water for various uses, including water supply and irrigation and for designing reservoir size. Given little observed streamflow data is available before the 1930s in the United States, studies focused on gaining insights on annual moisture supply prior to the observational period using paleo-information^1–4^. The high dependency between moisture availability and tree-ring chronologies have provided information on wet and dry periods by reconstructing streamflows over pre-instrumental periods^5–8^. While tree-ring chronologies have good skill in low flow estimation^9–11^, they have limitations in estimating high flows. Due to the upper bound on water holding capacity of soil, trees’ metabolic growth limits and increased cloud cover during wet years, high flows are not properly estimated using tree-ring chronologies^3,12–14^. Studies have shown that incorporating sea surface temperature (SSTs) along with tree-ring chronologies resulted in improved reconstruction of streamflow and wildfire synchrony^15–17^. Recent progress in understanding ocean-atmosphere interactions and the ensued teleconnection shows that there are well organized modes of interannual and interdecadal variability in climate that modulate these dominant moisture delivery pathways and has significant projections on continental scale rainfall and streamflow patterns^18,19^. Interannual modes such as the El Nino-Southern Oscillation (ENSO) resulting from sea surface conditions in the tropical Pacific Ocean primarily determine the interannual variability in precipitation over North America^20,21^. There are also other dominant decadal and interdecadal climatic modes such as Pacific Decadal Oscillation (PDO) and North Atlantic Oscillation (NAO) that putatively govern the interannual variability in climate over the North America^22^. To reduce the limitation of tree rings in reconstructing high flows^22^, proposed a hybrid reconstruction methodology using SST and tree-ring chronologies building upon the teleconnection signal with exogenous climatic conditions^22^. suggested separating the moisture contributed from exogenous climatic conditions (e.g., SSTs) as “outside-the-region moisture supply” and moisture contributed from the same hydroclimatic region as “within-the-region moisture supply”. Since the study^22^ focused on the Southeast US (SEUS), outside-the-region moisture transport was estimated by El-Nino Southern Oscillation (ENSO) Index, Nino3.4, the within-the-region moisture contribution was estimated by tree-ring chronologies. Given the influence of outside-the-region moisture supply on streamflow is separately estimated by exogenous SST conditions, it partially improves high flow estimation that is contributed by SST sources. The hybrid reconstruction approach, relying on utilizing multi-proxies (i.e., SST and tree-ring chronologies) for streamflow reconstruction, by^22^ improved overall skill and resulted in better above-normal flow estimates in comparison to the traditional tree-ring reconstructions in eight stations across the SEUS, which is a region with limited tree-ring chronologies. We perform here a national-scale hybrid streamflow reconstruction for 301 stream gauges (Fig. 1) that are significantly influenced by low-frequency oscillatory signals over the coterminous United States (CONUS). In this analysis the SST streamflow components are identified using regression while^22^ used Singular Spectrum Analysis (SSA) for the same. Given that SSA approach requires low-frequency mode identification for separating SST signals, such an approach will be difficult in the current study which considers PDO that has a periodicity of 16–20 years. The current approach is such simpler by explaining the role of SST predictors in influencing the Box-Cox transformed streamflow.

**Figure 2:**
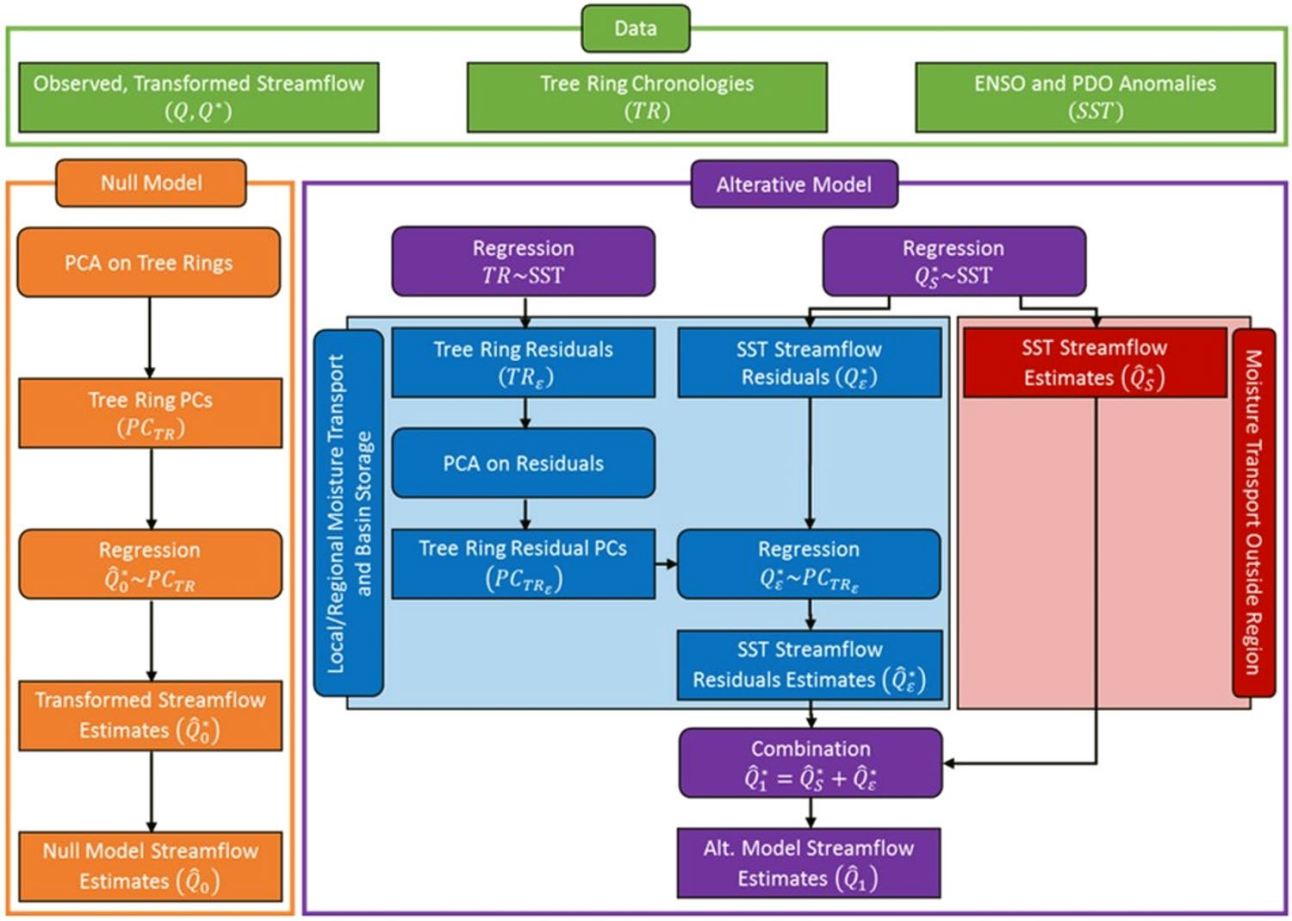
Data and framework for the two annual streamflow reconstruction, NM (orange) and AM (purple), considered in the study. Rectangles represent data and the operator ~ represents linear regression with the intercept model between the predictand (RHS of the operator) and the predictors (LHS).

Since the hybrid streamflow reconstruction is at the national scale, both ENSO and Pacific Decal Oscillation (PDO) conditions are used as SST conditions that have been shown to significantly affect moisture transport over the CONUS^21,23,24^. Details on the variability explained by ENSO and PDO on the annual flows of the selected Hydro-Climatic Data Network (HCDN) basins is given in Supplemental Information (SI) (Figs SI-1 and SI-2). For each HCDN basin, tree ring and SST predictors are chosen based on correlation, proximity and length of data (See Data and Methods (DM) and SI). Streamflow at each site is reconstructed using two models: a) Null model (NM) – the traditional principal components (PCs) based tree ring reconstruction model and (b) the Alternate Model (AM) – hybrid SST and tree ring reconstruction model. The NM reconstructs streamflow using a linear regression between PCs of the tree rings and the Box-Cox transformed flow (see DM) (Fig. 2 Orange). In contrast, the AM separates streamflow into components (Fig. 2 Purple) by regressing transformed streamflow against SSTs and regressing the residuals from the SST regression with the PCs of tree rings (Fig. 2 Blue). This is similar to the methodology in 36 but uses a much simpler approach in separating the moisture transport from outside the region (i.e., using SSTs) and local/regional moisture transport and basin storage (i.e., using SST and tree-ring chronologies) (See DM and SI).

**Figure 3:**
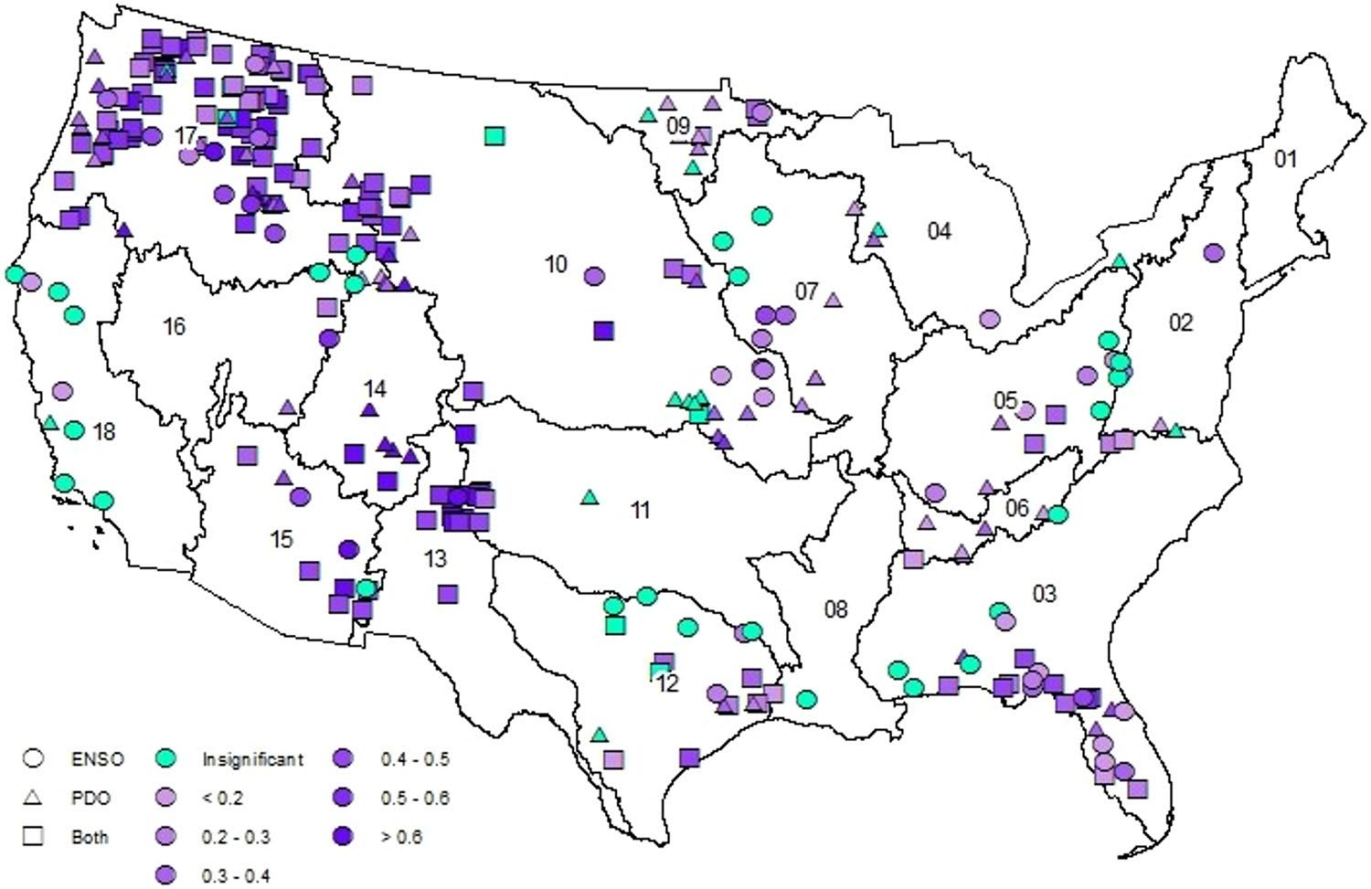
Adjusted R^2^ values for the AM in the selected 301 basins over the CONUS. Cyan color indicates the correlation between observed and estimated streamflow was insignificant by both AM and NM. This map is created using software suite ArcGIS 10.2.2 for Desktop, version number 10.2.2.3552 (http://www.esri.com/en/arcgis/products/arcgis-pro/overview).

**Figure 4:**
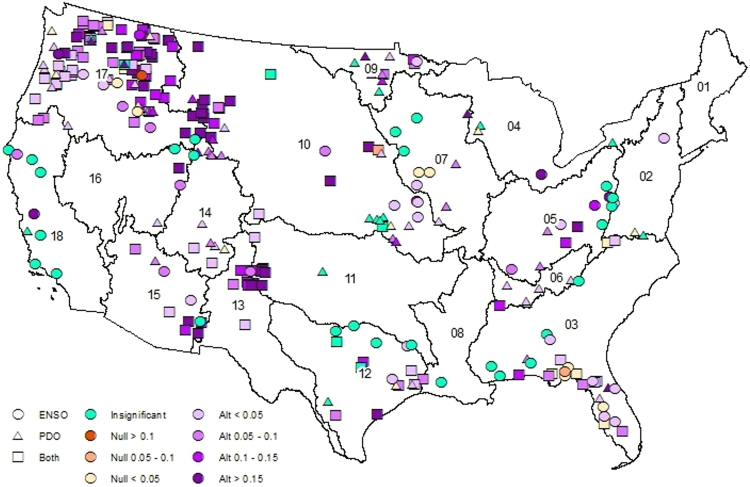
Comparison of adjusted R^2^ from NM and AM. Sites where AM (NM) has higher adjusted R^2^ indicated in darker purple (orange). Cyan indicates an insignificant correlation between estimated and observed streamflow by both models. This map is created using software suite ArcGIS 10.2.2 for Desktop, version number 10.2.2.3552 (http://www.esri.com/en/arcgis/products/arcgis-pro/overview).

**Figure 5:**
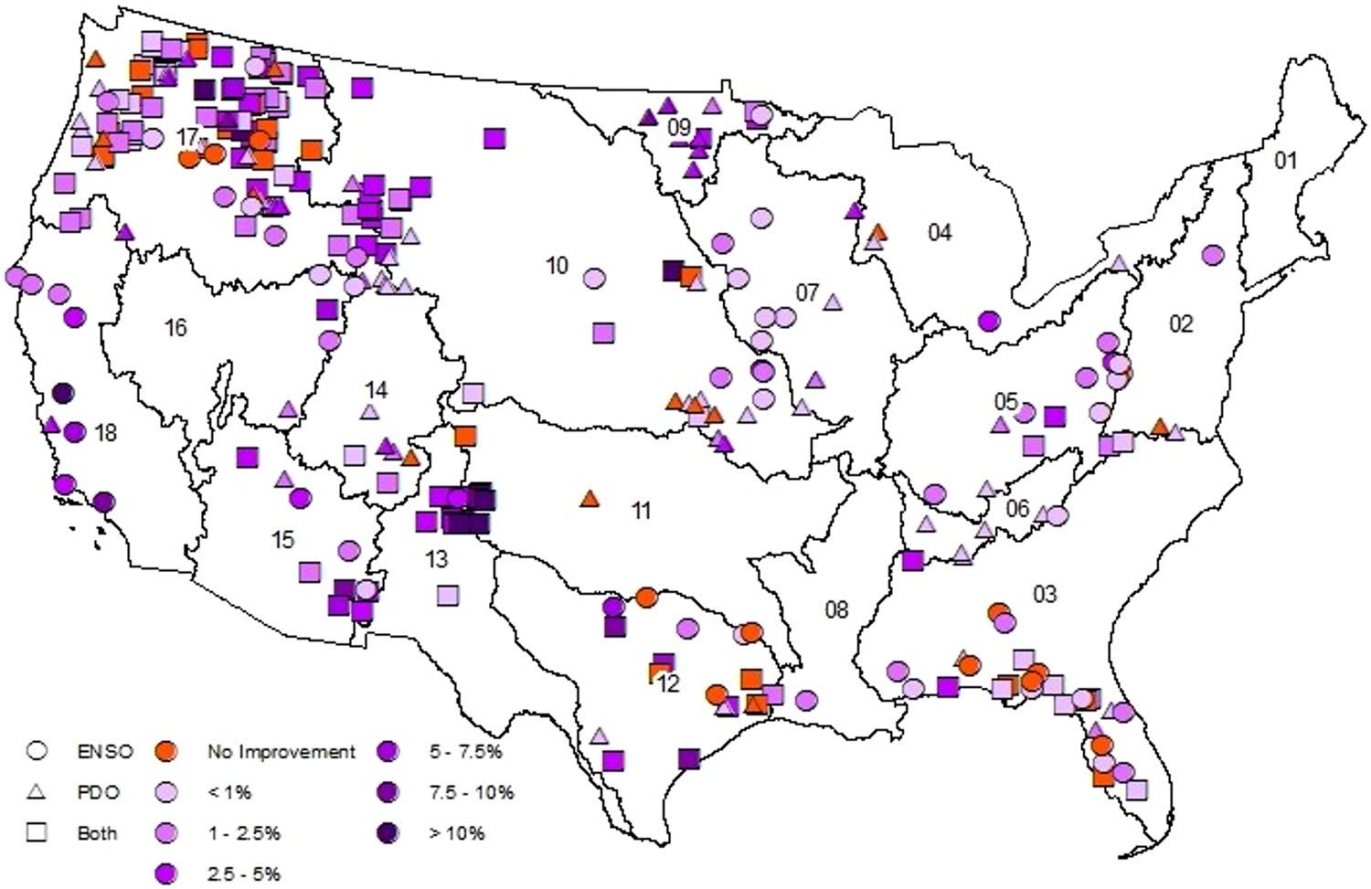
Comparison of above normal N-RMSE values of estimates from NM and AM. The improvement of above normal N-RMSE of AM from NM is indicated as in percentage of mean annual streamflow. Purple (orange) indicates AM (NM) had lower N-RMSE than NM (AM). This map is created using software suite ArcGIS 10.2.2 for Desktop, version number 10.2.2.3552 (http://www.esri.com/en/arcgis/products/arcgis-pro/overview).

**Figure 6:**
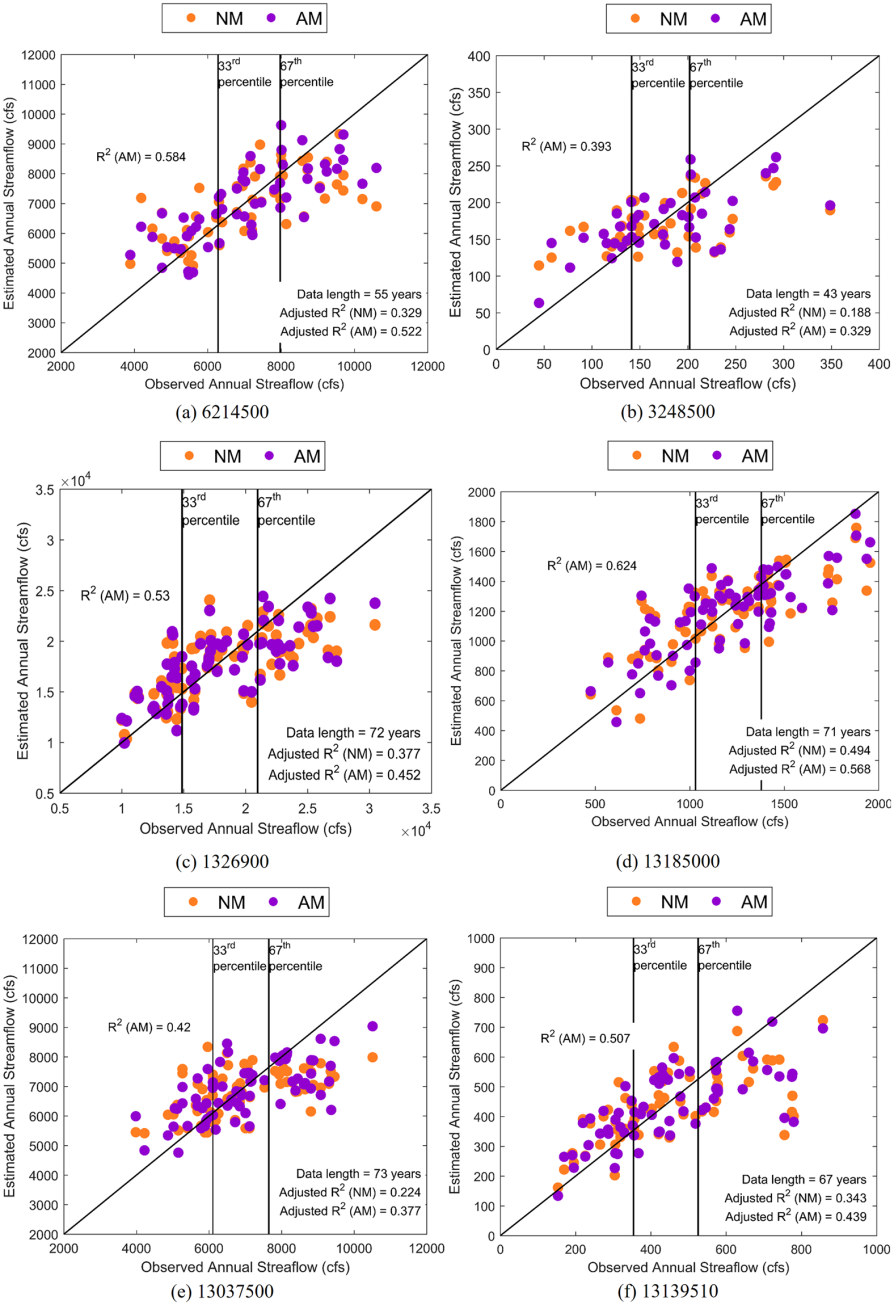
Comparison between observed and reconstructed annual streamflow for selected HCDN stations where AM shows good (>15%: **a,b**), moderate (5–10%: **c,d**), and marginal (<5%: **e,f**) improvements in adjusted R^2^ over NM. Terciles categories (below-normal <33rd percentile; above-normal >67th percentile; Normal – 33^rd^ to 67^th^ percentile of annual flows) of annual flows are also marked along with data length and adjusted R^2^ of the NM and AM.

Since the AM has more predictors than the NM, the skill of both models is compared using adjusted R^2^, ${\bar{R}}^{2}$, (See SI-3 for details) to understand the improved performance is due to the information in the additional predictors (i.e., SSTs) rather than due to addition of parameters^25^. Figure 3 shows AMs have significant ${\bar{R}}^{2}$ values for majority of HCDN basins over the CONUS, particularly for basins in the Pacific Northwest and Sunbelt regions, where the ENSO and PDO signals have strong influence^21,23,24^. Both models showed insignificant skill in predicting observed flows (cyan colored sites in Figs 3 and 4). Compared to NM ${\bar{R}}^{2}$ (Fig. 4), the AM shows a significant improvement over NM for majority of basins over the CONUS. Even in basins where the NM performed better, the difference in ${\bar{R}}^{2}$ was lesser than 0.05 (Table SI-1). But, the AM shows a significant improvement across the CONUS with the largest improvement being in the Pacific Northwest and Sunbelt regions, where the ENSO and PDO signal is greatest. In the Sunbelt east (Hydrologic Unit Code (HUC 2) regions 03, 08 and 12), the AM explains an additional 5–10% variability (i.e., difference in ${\bar{R}}^{2}$ between 0.05 and 0.1) over majority of the basins. Further, in Sunbelt west (HUC2 regions 11, 13 and 15) and Pacific Northwest (HUC 2 regions 10 and 17), the AM explains an additional 5–10% variability in almost all sites and has several sites where the ${\bar{R}}^{2}$ improvement exceeds 0.15. The AM also has lower Normalized Root Mean Square Error (N-RMSE: see SI-3 for details) than the NM in 268 (89%) basins (Fig. SI-3). While the NM performs better in 11% of basins, the average improvement in ${\bar{R}}^{2}$ is only 2% compared to 8% average improvement across basins where the AM performs better. In predicting above normal flows (Figs 5, SI-4), the AM performs better in 257 basins (85%) with an average improvement of 9% while the NM only performs better in 44 basins (15%) with an average improvement of 3%. In general, the improvements are better over the Sunbelt and Northwest. The reconstructed flows from the AM show good skill in predicting above-normal flows (i.e., above 67^th^ percentile of annual flows) (Fig. 6) and also improves the overall skill in reconstruction. For sites with NM performing better (Fig. SI-5), the improvements in predicting above-normal flows are only marginal resulting in reduced improvements in the overall ${\bar{R}}^{2}$. Further, NM mostly performs better for smaller basins with smaller mean annual discharge (Fig. SI-6). In smaller basins, it is natural to expect the role of large-scale climate teleconnection to be minimal as the basin scale hydroclimate dominates, thereby reduced role of SST in improving the reconstruction. Thus, most improvements on overall ${\bar{R}}^{2}$ comes from the improvements in reconstructing above-normal flows. Streamflow, being the response of the land surface, is controlled by other factors such as heterogeneity in forcings – precipitation and temperature – and spatial variability in soil and aquifer characteristics. Thus, the proposed hybrid approach improves streamflow reconstruction over the traditional approach, but still falls short in explaining the total variability due to spatial variability in forcings and land surface heterogeneity.

## Discussion

The proposed hybrid methodology emphasizes using multi-proxies for reconstructing streamflow. Several studies have improved the reconstruction of climate fields using paleo-information from multiple proxies^26–28^. Multi-proxies – sediment deposits and pre-observational flood information – have been used to reduce the uncertainty in flood frequency estimation^29,30^. Findings in this study are in line with 36 in demonstrating that including SST with tree rings for streamflow reconstruction improves above normal (Fig. 5) flows and in the process improves overall reconstruction skill (Figs SI-3, SI-4). Further, the ${\bar{R}}^{2}$ value comparison in Fig. 4 shows that the skill improvement is due to the signal in the SSTs and not due to the addition of parameters. This is consistent with other studies that have used SSTs to improve reconstructions in snow water equivalent^15,16^ and wildfires^17^, in demonstrating the utility of SST in reconstruction. This hybrid approach of using SSTs could potentially improve upon other reconstruction efforts such as precipitation^31^, extreme precipitation^32^ and drought indices^33–35^. In addition, the AM shows highest skill (Fig. 3) and greatest improvement (Fig. 4) over the NM in the Pacific Northwest and Sunbelt regions of the United States where low-frequency oscillations have strong influence in regional hydroclimate^21,23,24^. Thus, regions whose hydroclimatology exhibit significant association with low frequency oscillatory signals could consider including SST anomalies as additional predictors.

Though the reconstruction was performed in virgin basins with relatively long data records from the CONUS, the hybrid reconstruction methodology is expected to provide significant benefits for basins with limited streamflow records as both SST anomalies and tree-ring chronologies are available for a longer period. Further, both these data sources extend beyond the available meteorological records (i.e., precipitation), thereby ably supporting Prediction in Ungauged Basins (PUB) efforts^36^. In addition to ungauged basins, improved streamflow reconstructions can also benefit basins with significant anthropogenic influences (e.g. reservoir storage and groundwater pumping). For instance, existing naturalized inflow time series for basins with significant anthropogenic influences could be used with the proposed hybrid methodology for developing reconstructed flows, which could be used for capacity expansion of reservoir and for revising the current design yield considering the paleo-information. In fact, studies have shown the information from streamflow reconstruction improves reservoir sizing^37^ and provide better insight into future streamflow conditions in comparison to near-term climate change projections^38^. Potential to re-assess operational rule curves to incorporate environmental flows also exists using the augmented record^39,40^.

Although the proposed hybrid methodology shows improvements in reconstruction skill, the methodology has limitations. Since the model estimates the outside-the-region moisture transport using SST anomalies, the proposed reconstruction could be applied only for basins that exhibit significant influence due to low frequency climatic signals. In addition, while the SST data is of good quality for a significant period (1940 – present), SST data prior to 1940^41^ is primarily estimated from ships and limited buoys. Thus, additional investigation is needed on how the uncertainty in SSTs propagates into streamflow reconstruction. One approach is to use available meteorological records from 1900s with a hydrologic model and compare with the reconstructed flows. Another approach is to replace the SST anomalies with the General Circulation Model (GCM) simulated SST time series from Coupled Model Intercomparison Project Phase 5 (CMIP5)^42^ with the hybrid methodology and compare the performance with the AM skill. However, both these additional validation efforts suffer from errors from the hydrologic model and from GCMs.

Since the hybrid methodology considers SST data for reconstruction, it also depends on the length of the SST data, which is available from 1856 onwards. Thus, it potentially limits the length of reconstructed data. Dendrochronological studies have developed reconstructed hydroclimatic data using multi-species data having different uncertainty over different time intervals^35,43^. Similar nested reconstruction techniques could be considered to combine the reconstructed streamflows from the hybrid approach (i.e., post 1856 period) and the traditional approach (i.e., prior to 1856), thereby developing a long time series of reconstructed streamflows with different uncertainty over the entire period. Similarly, the proposed methodology also did not consider inter-site correlation as the effort here focused at the national scale. One could consider techniques^44^ that explicitly considers inter-site correlation with the proposed technique to accommodate for basins exhibiting significant inter-site correlation.

The inability of tree-ring chronology to capture the moisture availability during wet years due to soil and metabolic limits results in reduced interannual variability in the reconstructed annual streamflows. Given the limitation in quantifying wetter conditions, it is expected that the uncertainty in tree-ring chronologies to be larger in comparison to instrumental records in quantifying the moisture availability over the basin. So, our hybrid approach of using multi-proxies – SST and tree-rings combined – improves the streamflow reconstruction. We also considered other teleconnection indices such as North Atlantic Oscillation and Atlantic Multidecadal Oscillation in influencing the annual streamflows, but only fewer basins showed significant skill in influencing the annual streamflows. Studies^26^ have shown that both these indices influence the hydroclimatology of the basins predominantly at the regional level. Further, we did not consider it for reconstruction due to non-availability of these indices dating back to 1856. Extending these indices back to 1856 and incorporating them in the proposed hybrid methodology of using multi-proxies could further improve the reconstructed streamflows for basins exhibiting influence with AMO and NAO.

### Data and Methods

Streamflow data used for reconstruction methodology are part of the HCDN database^45,46^ and are considered as undeveloped/virgin basins with no impacts due to upstream storage or groundwater pumping. The tree-ring chronologies used for streamflow reconstruction in this study are from the National Atmospheric and Oceanic Administration (NOAA) International Tree Ring Data Bank (ITRDB) (Fig. 1). Tree ring chronologies were selected as predictors for a streamflow site if the tree-ring chronologies and HCDN gauge: (1) belong to the same water resources (HUC 2) region (Figs (1,2) are located within 400 km from each other and (3) have 20 or more years of common data (Fig. SI-2(b)). Only HCDN streamflow basins with at least two tree-ring chronologies identified were considered for this study.

Given the analysis focused at the national scale, both ENSO and PDO indices were considered as predictors^21,23,24^. ENSO conditions, denoted by Nino3.4, were obtained from 1856 from International Research Institute for Climate and Society (IRI) data library using the Kaplan’s SST^41^. Time series of PDO from 1856 was also obtained from the NCEP teleconnections database^47^ using Extended reconstructed SST (ERSSTv4)^48^. Both Kaplan’s and ERSSTv4 SST database utilize optimal interpolation methods to develop a unified global SST database with uncertainty bounds from 1856 to till date by combining SST observations from buoys and ships. For details see^41,48,49^. Basins were classified as ENSO and PDO affected if the Spearman’s rank correlation between the observed streamflow and Nino 3.4 and PDO indices respectively was significant at a 95% confidence level, and HCDN basins affected by neither were not considered for this study. Correlations between Nino 3.4 and PDO and annual streamflow are shown (Figs SI-1 and SI-2) in SI. Out of the 301 stations, 69 (85) were affected by ENSO (PDO) and 147 were impacted by both ENSO and PDO.

Figure 2 presents the reconstruction methodology for the AM and NM. Similar to 36, the hybrid reconstruction model separates streamflow into components of moisture transport from outside the region, which will be explained by SST, and local/regional moisture transport and basin storage, which will be explained by tree ring chronologies. Both reconstruction models utilize linear regression, so the Box-Cox transformed predictands follow a normal distribution. Using Lilliefors test^50,51^, the normality of the Box-Cox -transformed flows at each site was first tested based on a 95% significance level (Fig. SI-7). [see SI for additional information and Fig. SI-8 for lambda values] was performed and the transformed time series was used as a predictand in Fig. 2. The proposed hybrid reconstruction methodology used in this study is similar to the reconstruction methodology presented in^22^, which separate streamflow into moisture transport from outside the region, estimated by SST anomalies, and local moisture transport and basin storage, estimated by tree-ring chronologies after SST signal using Singular Spectrum Analysis (SSA)^52^. SSA is a dimension reduction analysis which identifies periodic components of a time series by comparing lagged versions of the time series^52^. only considered the ENSO signal on streamflow which has a periodicity of three to seven years. In contrast, PDO has a periodicity of 10–20 years, making the signal difficult to identify, especially for HCDN basins with less than 30 years of data. Given the complications of PDO in this study, we pursued the approach presented in Fig. 2 for quantifying outside-the region moisture transport.

### Null Model methodology

The NM (orange) utilizes only tree-ring chronologies identified as predictors for each site to explain all components of annual streamflow. Since tree ring chronologies across the sites exhibit high correlation, principal component analysis (PCA) was performed on the tree ring chronologies to obtain predictors for the regression model^4,7,53^. Using tree-ring principal components (PCs) explaining 90% of the variance and the transformed streamflow (${Q}_{0}^{\ast })$, linear regression^54^ coefficients along with the intercept are estimated for the NM (${Q}_{0}^{\ast } \sim P{C}_{TR}$ – the operator ~ represents the linear regression model with the intercept) resulting in transformed streamflow estimates $({\hat{Q}}_{0}^{\ast })$. The NM reconstructed streamflow $({\hat{Q}}_{0})$ are obtained by inverse of the transformation (log or Box-Cox) used for the site.

### Alternative Model Methodology

AM (purple) (Fig. 2) separates the outside the region moisture transport and the local/regional moisture transport and basin storage components. Components are identified and separated by linearly regressing the transformed streamflow against the identified SST anomaly(s) as described previously $({Q}_{s}^{\ast } \sim SST)$. Using the linear regression coefficients, the SST-explained streamflow $({\hat{Q}}_{s}^{\ast })$ and SST streamflow residuals $({Q}_{\varepsilon }^{\ast })$ are assumed as the moisture transport from outside the region (red) and the local/regional moisture transport and basin storage (blue) components of streamflow respectively. Since tree ring chronologies also have the signal from the moisture transport from outside the region, SST influence on chronologies is removed by regressing each tree ring chronology against the SST anomaly(s) identified for the basin $(TR \sim SST)$ and the resulting residuals $({\varepsilon }_{TR})$ are retained for PCA Similar to the NM, these residuals from different tree ring chronologies will be highly correlated, so a PCA is performed and the components explaining 90% of variance is retained $(P{C}_{T{R}_{\varepsilon }})$. The SST streamflow residuals $({Q}_{\varepsilon }^{\ast })$ are then regressed with the retained PCs $({Q}_{\varepsilon }^{\ast } \sim P{C}_{T{R}_{\varepsilon }})$ to obtain the within basin streamflow component $({\hat{Q}}_{\varepsilon }^{\ast })$. The AM transformed streamflow estimates $({\hat{Q}}_{1}^{\ast })$ are calculated by adding the SST streamflow estimates $({\hat{Q}}_{s}^{\ast })$ and residual estimates $({\hat{Q}}_{\varepsilon }^{\ast })$. Finally, the AM streamflow estimates $({\hat{Q}}_{1})$ are also obtained by transforming the transformed streamflow estimates $({\hat{Q}}_{1}^{\ast })$ back to the original space using the inverse of the log or Box-Cox transformation.

### Data availability

The datasets generated during and/or analyzed during the current study are available from the corresponding author on reasonable request.

## Electronic supplementary material

Supplementary information file
