# Supplementary material for: Role of Pacific SSTs in improving reconstructed streamflow over the coterminous US
Source: Sci Rep. 2018 Mar 21;8:4946. doi: 10.1038/s41598-018-23294-6 (PMC5862983; doi:10.1038/s41598-018-23294-6)
Supplement: Supplementary file 1 — Supplementary information file [file 41598_2018_23294_MOESM1_ESM.docx]

**Role of Pacific SSTs in improving reconstructed streamflow over the coterminous US**

**Sudarshana Mukhopadhyay, Jason M. Patskoski and A. Sankarasubramanian***

Department of Civil, Construction and Environmental Engineering

North Carolina State University

Raleigh, NC 27695-7908

***** - Corresponding author’s email: [sankar_arumugam@ncsu.edu](mailto:smukhop2@ncsu.edu)

(Submitted to Scientific Reports)

**Supplementary Information**

Table SI-1: Locations and descriptions of streamflow sites where the null model showed higher skill than the alternative model

| **USGS Station number** | **Location** | **Drainage Area [mi^2^]** | **Mean annual streamflow [cfs]** | **Number of tree rings** | **Adjusted R^2^ (Null model)** | **Adjusted R^2^ (Alt. model)** | **SST used in alternative model** |
| --- | --- | --- | --- | --- | --- | --- | --- |
| 02035000 | JAMES RIVER AT CARTERSVILLE, VA | 6257 | 7045 | 15 | 0.07 | 0.07 | PDO |
| 02053800 | S F ROANOKE RIVER NEAR SHAWSVILLE, VA | 110 | 112 | 7 | 0.25 | 0.25 | Both |
| 02298830 | MYAKKA RIVER NR SARASOTA, FLA. | 229 | 248 | 8 | 0.18 | 0.18 | Both |
| 02303000 | HILLSBOROUGH RIVER NR ZEPHYRHILLS, FLA. | 220 | 235 | 9 | 0.15 | 0.14 | ENSO |
| 02326000 | ECONFINA RIVER NEAR PERRY, FLA. | 198 | 138 | 15 | 0.47 | 0.46 | Both |
| 02327100 | SOPCHOPPY R NR SOPCHOPPY, FL | 102 | 182 | 16 | 0.37 | 0.37 | ENSO |
| 02327500 | OCHLOCKONEE RIVER NEAR THOMASVILLE, GA. | 550 | 506 | 17 | 0.18 | 0.17 | ENSO |
| 02329000 | OCHLOCKONEE RIVER NR HAVANA, FLA. | 1140 | 1031 | 16 | 0.31 | 0.24 | ENSO |
| 02359000 | CHIPOLA RIVER NR ALTHA, FLA. | 781 | 1478 | 15 | 0.22 | 0.21 | Both |
| 02369800 | BLACKWATER RIVER NEAR BRADLEY AL | 88 | 141 | 8 | 0.11 | 0.10 | ENSO |
| 04078500 | EMBARRASS RIVER NEAR EMBARRASS, WI | 384 | 292 | 7 | 0.03 | 0.03 | PDO |
| 04081000 | WAUPACA RIVER NEAR WAUPACA, WI | 265 | 239 | 7 | 0.38 | 0.37 | PDO |
| 05280000 | CROW RIVER AT ROCKFORD, MN | 2520 | 897 | 12 | 0.02 | 0.02 | ENSO |
| 05451500 | IOWA RIVER AT MARSHALLTOWN, IA | 1564 | 946 | 19 | 0.40 | 0.39 | ENSO |
| 05470000 | SOUTH SKUNK RIVER NEAR AMES, IA | 315 | 192 | 17 | 0.42 | 0.41 | ENSO |
| 06481500 | SKUNK CR AT SIOUX FALLS SD | 622 | 103 | 7 | 0.38 | 0.31 | Both |
| 06884200 | MILL C AT WASHINGTON, KS | 344 | 99 | 4 | -0.04 | -0.07 | PDO |
| 06885500 | BLACK VERMILLION R NR FRANKFORT, KS | 410 | 165 | 6 | 0.13 | 0.13 | PDO |
| 06889200 | SOLDIER C NR DELIA, KS | 157 | 91 | 10 | 0.43 | 0.39 | PDO |
| 07234000 | BEAVER RIVER AT BEAVER, OK | 7955 | 11 | 5 | 0.18 | 0.16 | PDO |
| 08070500 | CANEY CREEK NR SPLENDORA, TX | 105 | 80 | 6 | 0.31 | 0.30 | PDO |
| 08101000 | COWHOUSE CREEK AT PIDCOKE, TX | 455 | 97 | 8 | 0.03 | 0.03 | Both |
| 09352900 | VALLECITO CREEK NEAR BAYFIELD, CO. | 72 | 143 | 35 | 0.78 | 0.78 | PDO |
| 09430600 | MOGOLLON CREEK NEAR CLIFF, NM | 69 | 30 | 7 | 0.02 | 0.01 | ENSO |
| 12039500 | QUINAULT RIVER AT QUINAULT LAKE, WASH. | 264 | 2878 | 43 | 0.35 | 0.34 | PDO |
| 12304500 | YAAK RIVER NEAR TROY, MT. | 766 | 842 | 27 | 0.23 | 0.21 | PDO |
| 12445000 | OKANOGAN RIVER NEAR TONASKET, WASH. | 7260 | 2950 | 53 | 0.31 | 0.31 | Both |
| 13235000 | SF PAYETTE RIVER AT LOWMAN ID | 456 | 849 | 27 | 0.57 | 0.55 | ENSO |
| 13310500 | S FK SALMON RIVER NR KNOX ID | 92 | 146 | 27 | 0.67 | 0.64 | PDO |
| 13331500 | MINAM RIVER AT MINAM,OR | 240 | 456 | 57 | 0.69 | 0.69 | ENSO |
| 13334700 | ASOTIN CR BLW KEARNEY GULCH NR ASOTIN, WASH. | 170 | 72 | 44 | 0.66 | 0.66 | Both |
| 13338500 | S FK CLEARWATER RIVER AT STITES ID | 1150 | 989 | 17 | 0.50 | 0.35 | ENSO |
| 14198500 | MOLALLA R AB PC NR WILHOIT, OREG. | 97 | 531 | 59 | 0.49 | 0.45 | PDO |


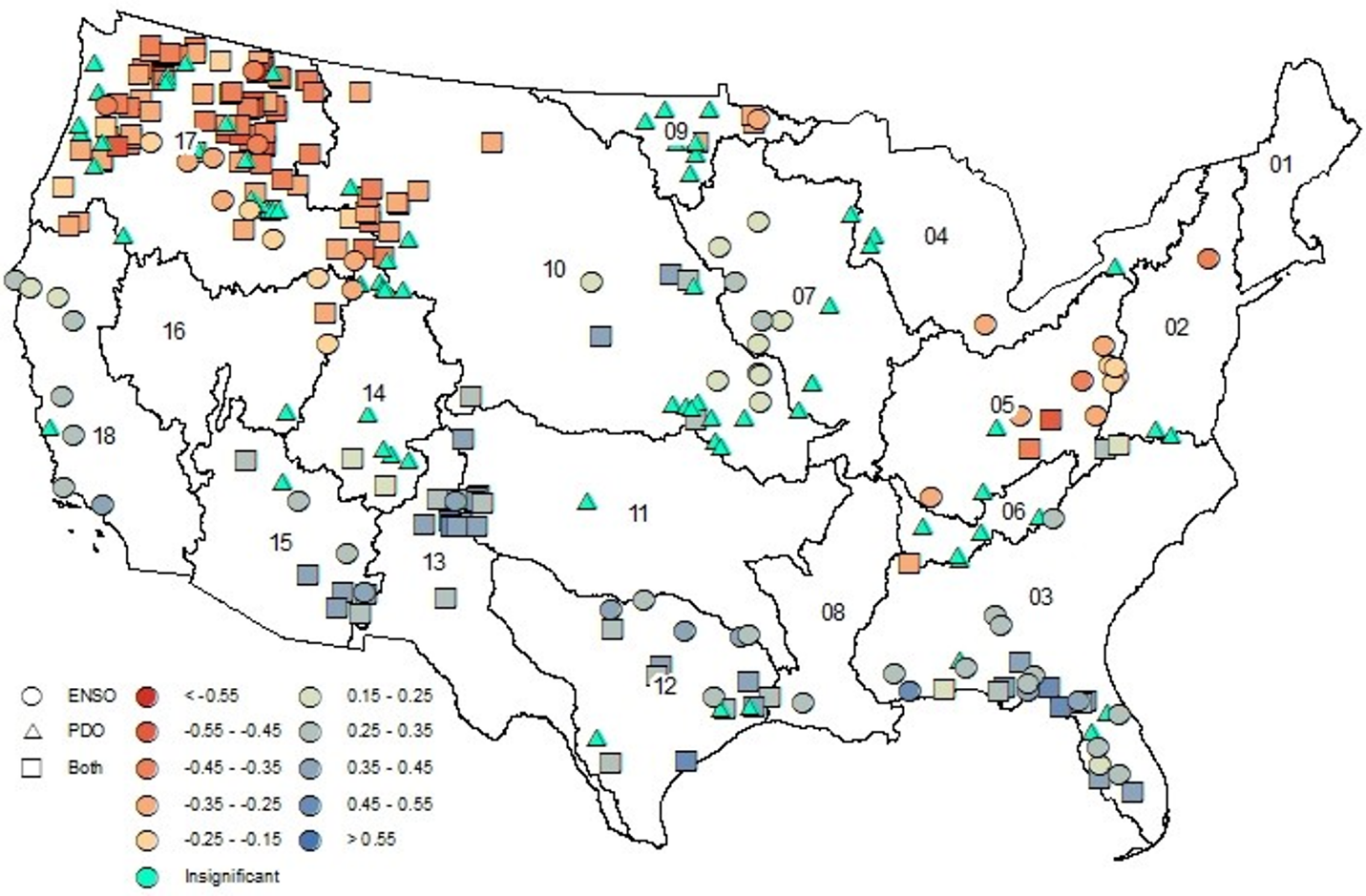


Figure SI-1: Spearman rank correlation between annual streamflow and ENSO (Nino 3.4 Index) for the basins used in this study. Insignificant correlation with observed flows are denoted with Cyan color, ENSO (PDO) affected basins are indicated with circles (triangles) and basins affected by both are indicated with squares. This map is created using software suite ArcGIS 10.2.2 for Desktop, version number 10.2.2.3552 (url: <http://www.esri.com/en/arcgis/products/arcgis-pro/overview> )


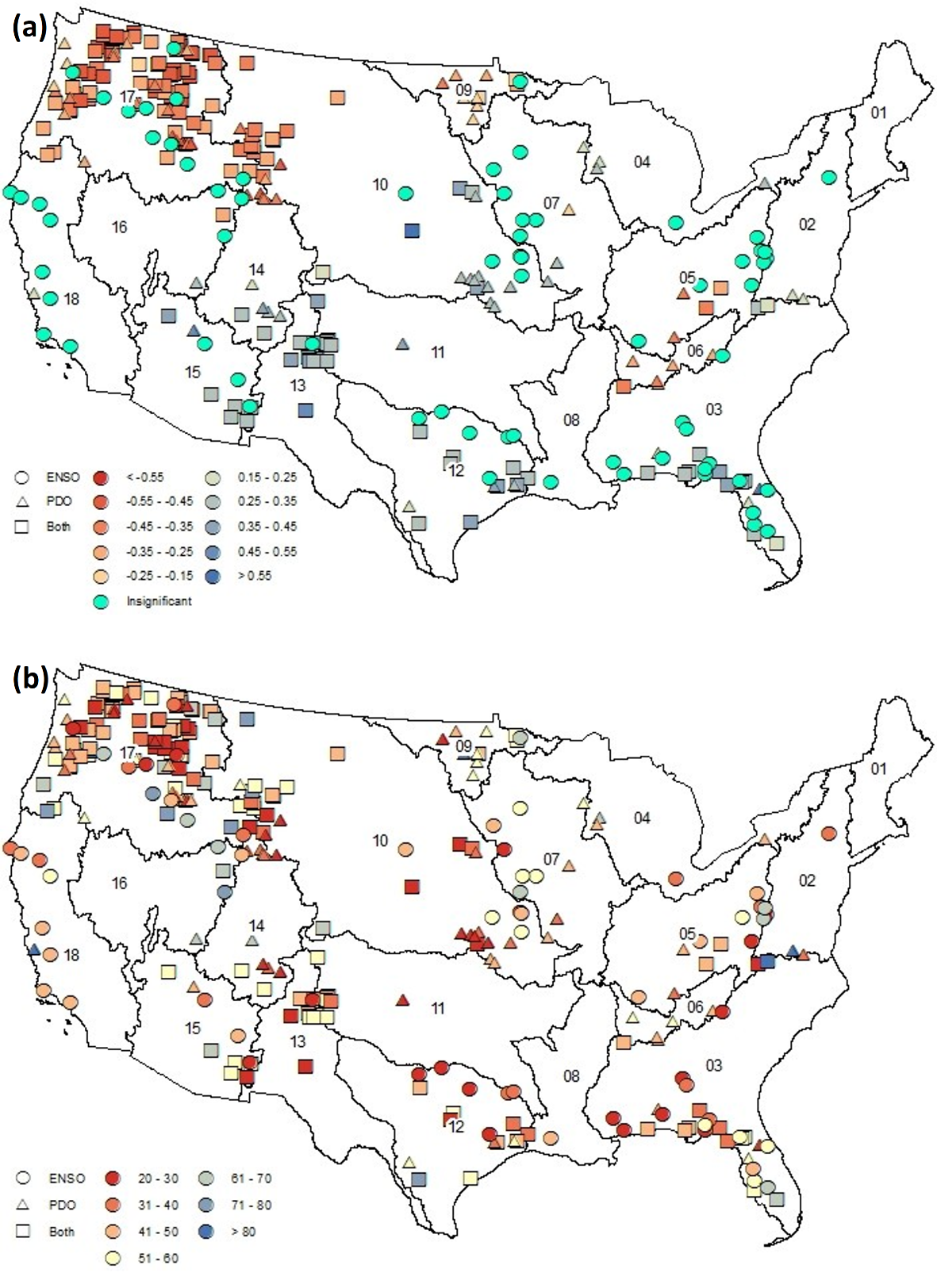


Figure SI-2: Role of selected predictors in influencing the annual hydroclimatology of the basin. (a) Spearman rank correlation between annual streamflow and PDO for the basins used in this study. Insignificant correlations are denoted with cyan color, ENSO (PDO) affected basins are indicated with circles (triangles) and basins affected by both are indicated with squares. Figure (b) the number of common years of annual streamflow, tree-ring chronology and SST data for each basin considered in this study. This map is created using software suite ArcGIS 10.2.2 for Desktop, version number 10.2.2.3552 (url: <http://www.esri.com/en/arcgis/products/arcgis-pro/overview> )


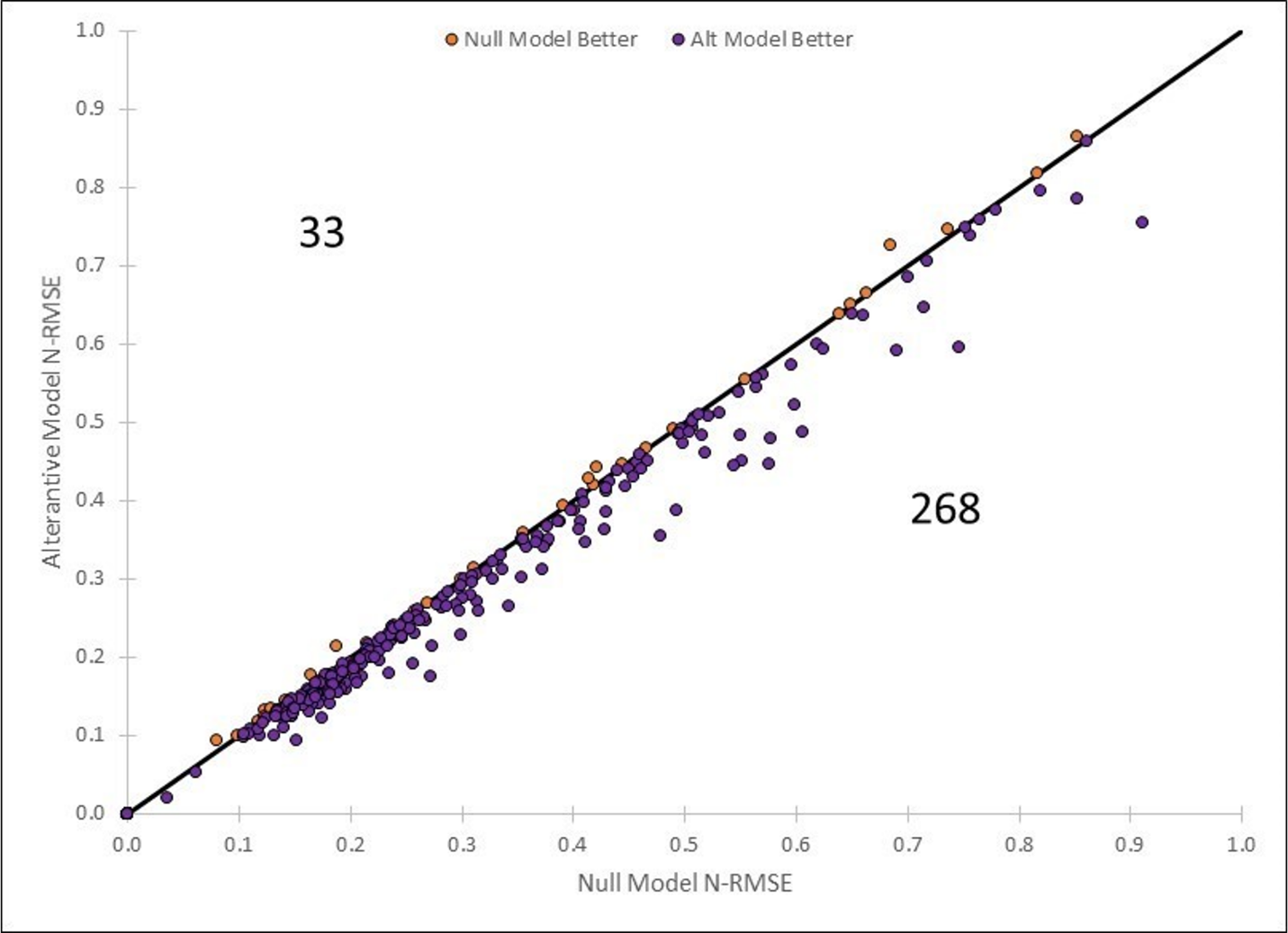


Figure SI-3: Comparison of N-RMSE values of estimates from NM and AM. Sites where the AM (NM) has the N-RMSE are indicated in purple (orange), and the number of sites where each model performed best is indicated in the figure.


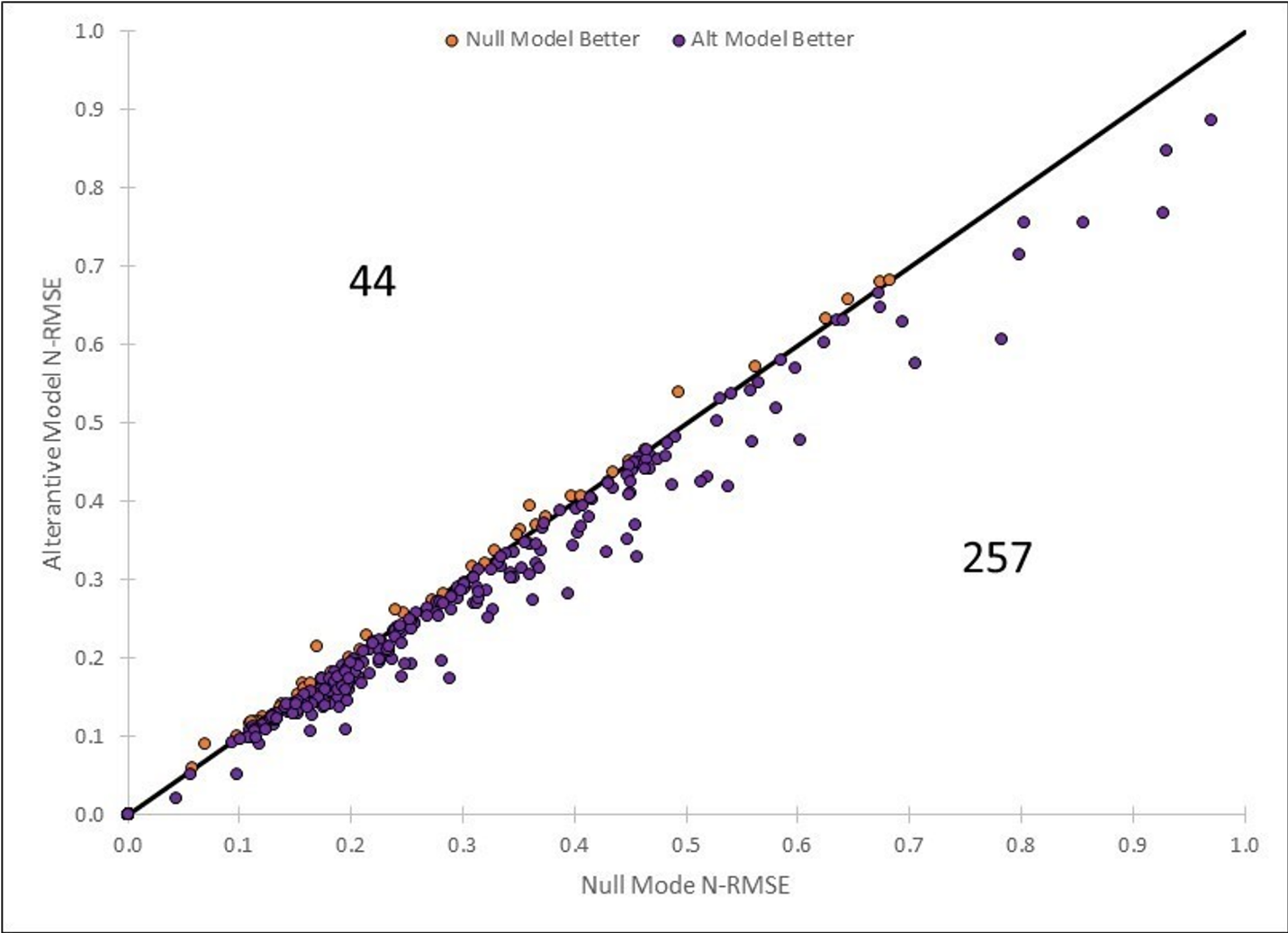


Figure SI-4: Comparison of above normal N-RMSE values of estimates from AM and NM. Sites where the AM (NM) has the lower above normal N-RMSE are indicated in purple (orange), and the number of sites where each model performed the best is indicated in the figure.


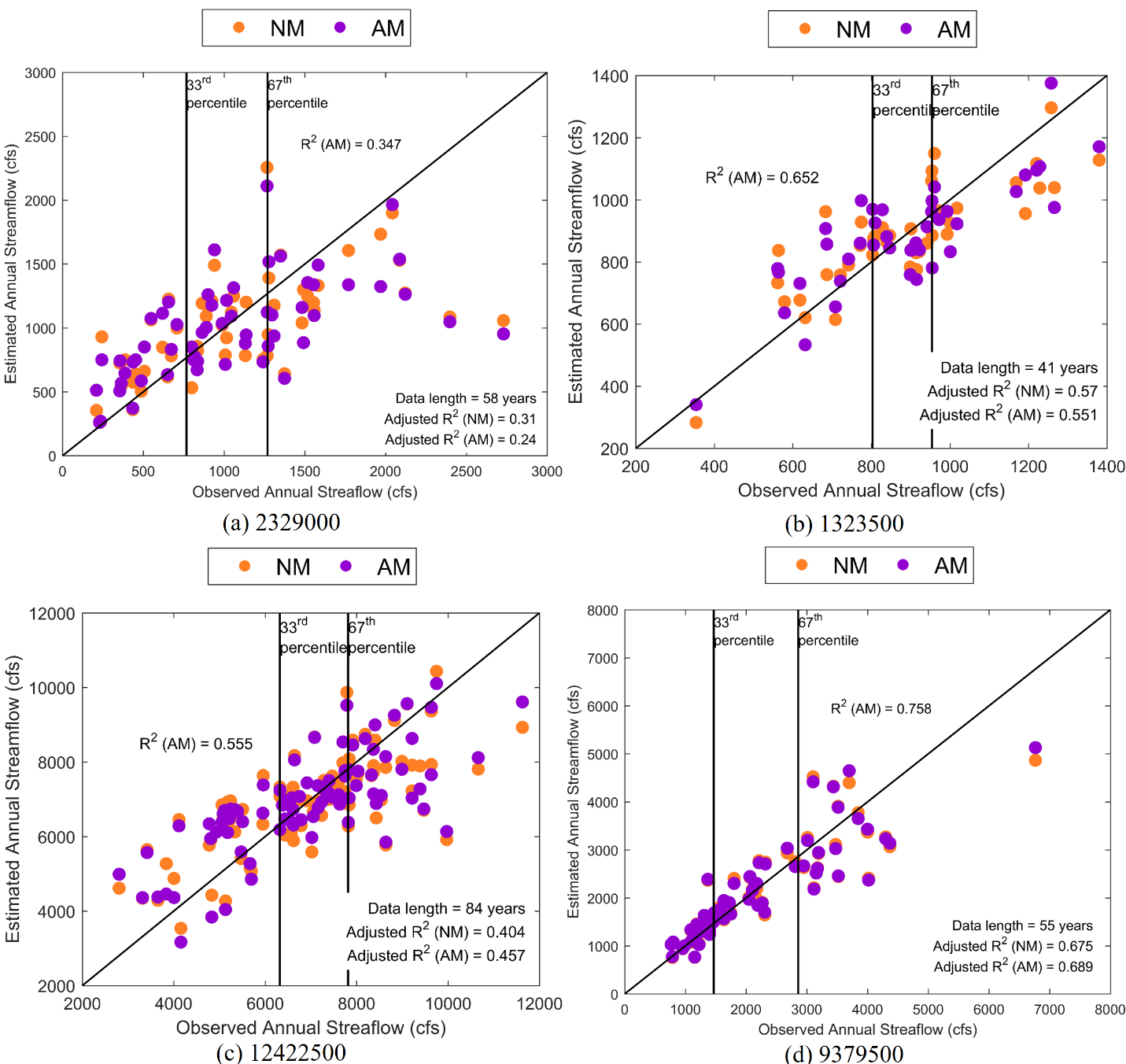


Figure SI-5: Comparison between observed and estimated annual streamflow for four selected HCDN stations where NM performs better than AM. R^2^ for AM is shown for each station.


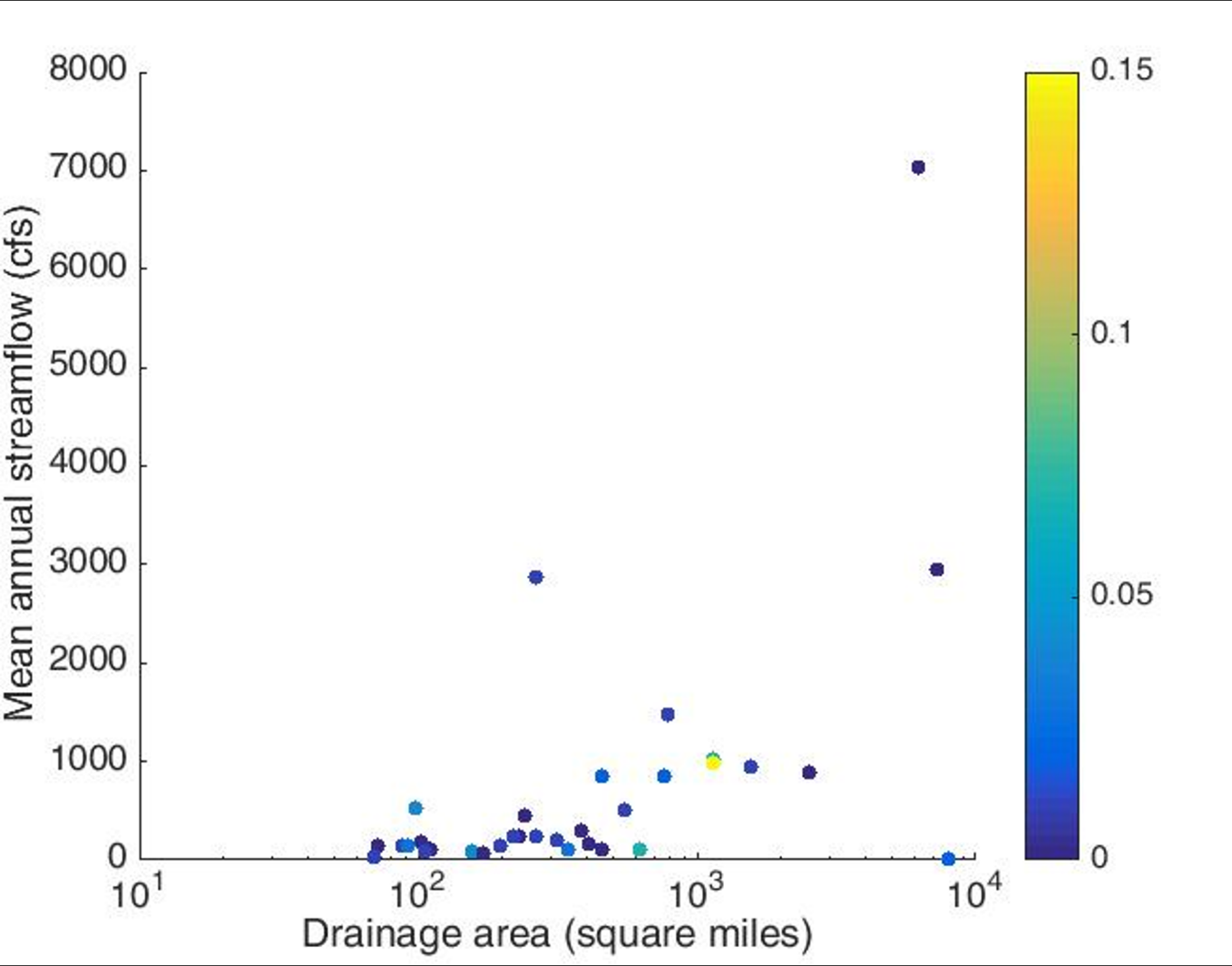


Figure SI-6: Drainage area and mean annual streamflow for basins where the NM (33 sites) has higher adjusted R^2^ values than the AM with the reconstructed flows from NM exhibiting significant skill with the observed flows. Differences in adjusted R^2^ values are labeled in colors.


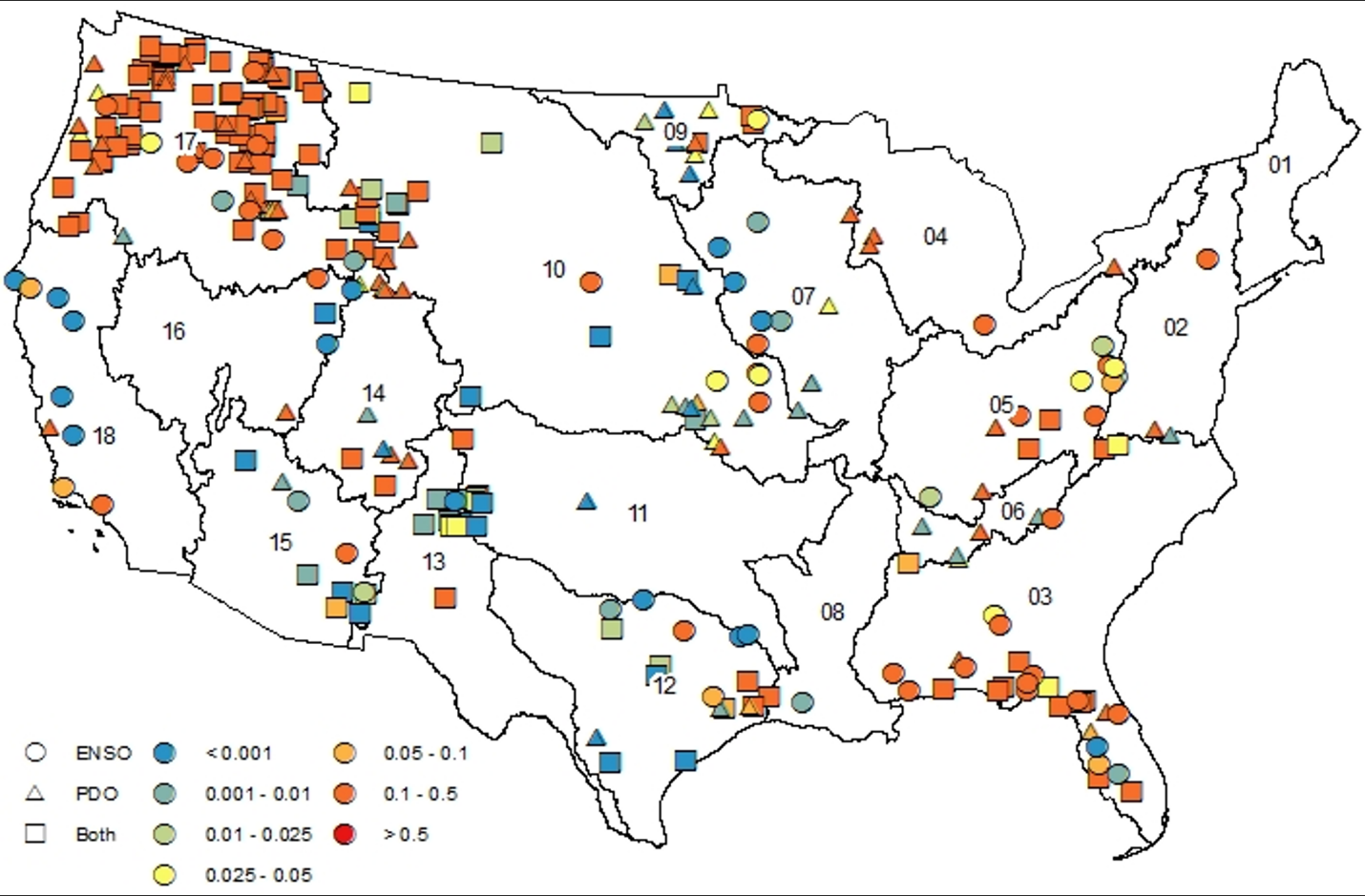


Figure SI-7: *p*-values for the Lilliefors test of normality on the annual streamflow. This map is created using software suite ArcGIS 10.2.2 for Desktop, version number 10.2.2.3552 (url: <http://www.esri.com/en/arcgis/products/arcgis-pro/overview> )


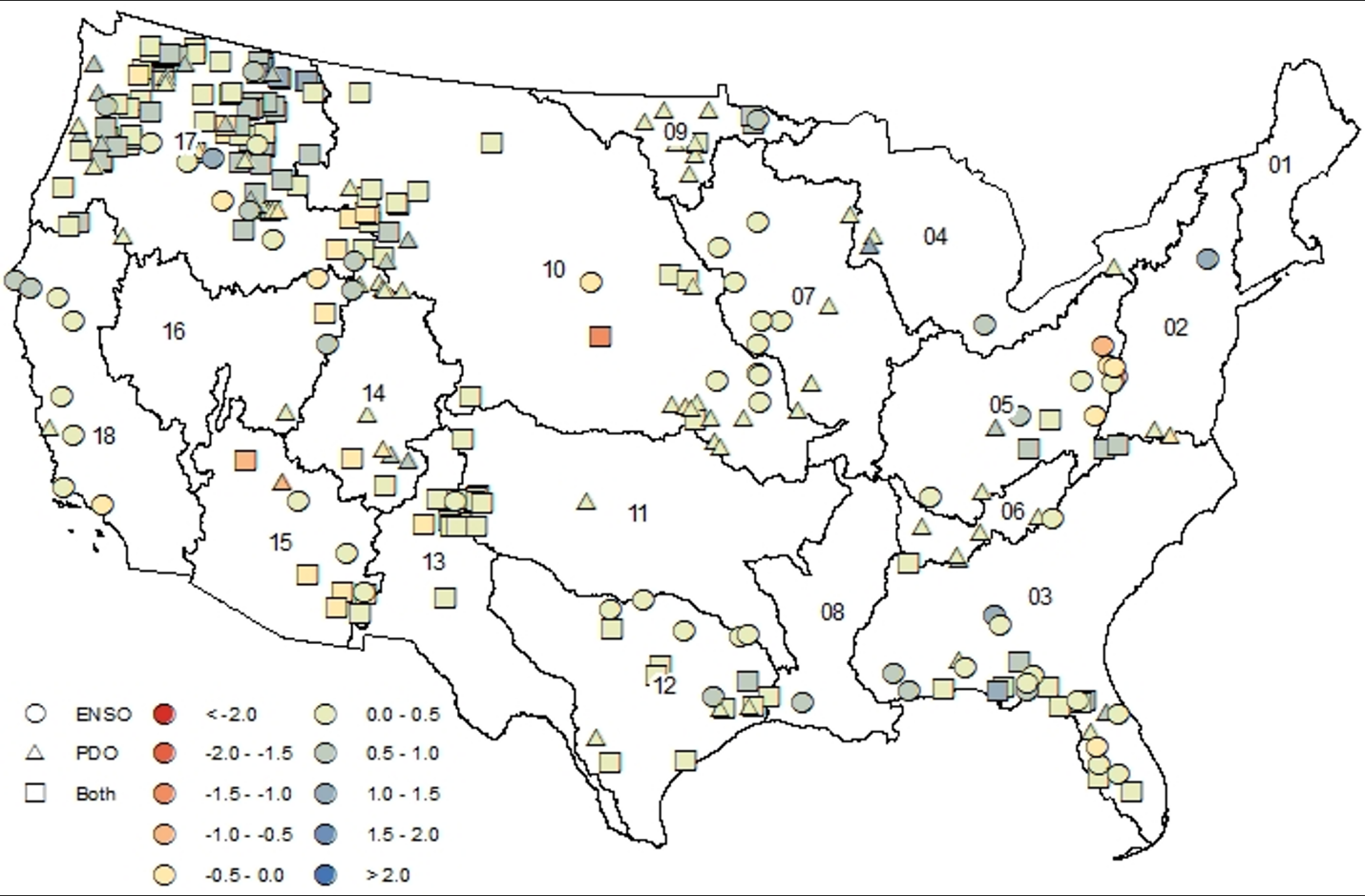


Figure SI-8: Lambda $(\lambda)$ values for Box-cox transformation. This map is created using software suite ArcGIS 10.2.2 for Desktop, version number 10.2.2.3552 (url: <http://www.esri.com/en/arcgis/products/arcgis-pro/overview> )

**Supplemental Information**

**SI-1.0 Correlation with ENSO and PDO**

As described in the main text, the AM reconstruction methodology utilizes SST anomalies to identify and predict the streamflow components resulting from moisture transport from outside the region. At the national level, outer region moisture transport is caused by both ENSO and PDO. Thus, each basin in this study will use Nino 3.4 or PDO or both Nino3.4 and PDO as predictors in the AM (Figure 1). The effect of both ENSO and PDO on annual streamflow is tested by computing Spearman rank correlation (Figures SI-1 and SI-2). Spearman rank correlation was chosen over Pearson correlation as it is more robust in quantifying the dependency under monotonic relationship between the predictors and predictand. The SST effects are most evident in the Pacific Northwest (HUC 2 regions 10 and 17) and Sunbelt regions (HUC 2 regions 03, 08, 11, 12, 13 and 15) of the CONUS. Both ENSO (Figure SI-1) and PDO (Figure SI-2) have negative correlation with annual streamflow in the Pacific Northwest and positive correlation with annual streamflow in the Sunbelt. The role of PDO in the Pacific Northwest is more pronounced than ENSO in the number of basins significantly affected (number of cyan circles vs triangles) and strength of signal (darkness of orange shapes). Alternatively, ENSO has a larger role, in both the affected basins and the signal strength, than PDO in the Sunbelt. However, it is important to note that more basins were affected by both PDO and ENSO (147) than by ENSO alone (69) or by PDO alone (85). In basins affected by both ENSO and PDO (squares in Figures SI-1 and SI-2), PDO has a stronger correlation in Pacific Northwest basins while ENSO has a stronger correlation in Sunbelt basins. The relationship between ENSO, PDO and annual streamflow in the CONUS shown in this study is consistent with previous studies on SST anomalies [[4]; [8]; [11]].

**SI-2.0 Basin Details and Predictor Identification Methodology**

This study evaluates two streamflow reconstruction models, so the streamflow data used must be consistent for the entire calibration/validation period. Thus, only streamflow data from the Hydroclimatic Data Network (HCDN) [[10][11] ] was considered in this study. HCDN basins are void of upstream storage and groundwater pumping and are considered as undeveloped/virgin basins. Using HCDN basins ensures that streamflow conditions remain the same for the calibration/validation as well as for the reconstruction (pre-observational) period. Given this study aims to reconstruct streamflow using both tree ring chronologies and SST anomalies, all chosen HCDN basins must have a significant Spearman rank correlation with Nino 3.4, PDO or both (Figures SI-1 and SI-2a). While only two SST anomalies are considered in this study, many tree ring chronologies are available across the CONUS (Figure 1), so unique tree-ring chronology predictors must be chosen for each HCDN basin. Similar to SST anomaly predictor identification, a tree-ring chronology must have a significant Spearman rank correlation with annual streamflow to be chosen as a predictor for the HCDN basins. However, to ensure the correlation between the tree-ring chronology and annual streamflow is due to the same climatic signal, the tree-ring chronology also must lie within the same water resources region (HUC-2) as the HCDN basin to be used as predictor.

For a fair model comparison, at least 20 years of common streamflow, SST anomaly and tree-ring chronology data are needed to calibrate and evaluate model performance. Both Nino 3.4 and PDO data are available from 1856 to the present, which completely overlaps with HCDN streamflow data (usually available from around 1930). However, tree ring chronology data is only available from the year the chronology was obtained to the beginning of the life of the tree. While most tree ring chronologies begin before 1856, tree ring chronologies have been collected since the 1950s. Thus, typically the oldest year of common of data will be the first year of recorded annual streamflow and the most recent year of common data will be the year of record of the oldest tree ring chronology for each HCDN basin. Consequently, the number of years of data available for modeling are the years within this range. Since this study requires at least 20 years of common data for model calibration, evaluation and comparison, a tree-ring chronology was not considered as a potential predictor if it did not have 20 years of common data with a HCND basin. Therefore, all HCDN basins used in this study have at least 20 common years of annual streamflow, tree-ring chronology and SST data (Figure SI-2b).

**SI-3.0 Reconstruction Methodology**

The proposed reconstruction methodology used in this study is similar to the reconstruction methodology presented in [9]. Both methodologies separate streamflow into moisture transport from outside the region, estimated by SST anomalies, and local moisture transport and basin storage, estimated by tree-ring chronologies after SST signal has been removed. The major difference between the two methodologies is the SST streamflow component is identified using Singular Spectrum Analysis (SSA) in [9], while in this analysis the SST components are identified using regression. SSA is a dimension reduction analysis which identifies periodic components of a time series by comparing lagged versions of the time series. [9] only considered the ENSO signal on streamflow which has a periodicity of three to seven years. In contrast, PDO has a periodicity of 10 – 20 years, making the signal difficult to identify, especially for HCDN basins with less than 30 years of data. Given the complications of PDO in this study, a different approach of identifying the streamflow component due to moisture transport from outside the region is considered.

The regressions in both the NM and AM use streamflow that has been log or box-cox transformed. Information on these transformations is given in SI-5.0 and SI-6.0. As described in the main text the NM (Figure 2 Orange) in this study is the traditional tree ring reconstruction approach. For a given HCDN basin, a PCA is performed on the identified tree-ring chronologies (SI-2.0), and components which explain 90% of the variance are retained $\left( {PC}_{TR} \right)$. Linear regression coefficients are then estimated using the PCs as predictors to estimate the transformed streamflow$\left( Q_{0}^{*}\sim{PC}_{TR} \right)$. Transformed streamflow estimates from the regression $\left( \hat{Q}_{0}^{*} \right)$ are brought back to the original space using the inverse of the original transformation for that HCDN basin to obtain NM streamflow reconstruction estimates$\left( \hat{Q}_{0} \right)$. Assuming $m_{0}$number of principal components of tree rings are retained, then the total number of parameters considered including the intercept term under NM is $(m_{0}+1)$for estimating reconstructed flows.

The AM (Figure 2 Purple) is slightly more involved by separating the streamflow component from moisture transport from outside the region (red) from the components from local/regional moisture transport and basin storage (blue). These components are identified by regressing the transformed streamflow with the identified SST anomaly for that HCDN basin$(Q_{s}^{*}\sim SST)$. The number of predictors under this regression is 1 (2) if only ENSO/PDO (both ENSO and PDO) influences. Accordingly, the number of parameters under this regression that quantifies exogenous moisture transport is 2 or 3 depending on the role of ENSO and PDO. The estimates from this regression $\left( \hat{Q}_{s}^{*} \right)$ are classified as the streamflow components due to moisture transport from outside the region, while the residuals from the regression $\left( Q_{\varepsilon}^{*} \right)$ are classified as the streamflow components due to local and regional moisture transport and basin storage. These residuals can be explained by the identified tree ring chronologies for the HCDN basin, but the tree ring chronologies also have components due to moisture transport from outside the region which need to be removed. Similar to the process for identifying the streamflow components due to moisture transport from outside the region, each tree ring chronology identified as a predictor for the HCDN basin is regressed against the same SST as the streamflow$\left( TR\sim SST \right)$. The residuals from this regression $\left( \varepsilon_{TR} \right)$ only have components of local and regional transport and basin storage, so they can be used to estimate the corresponding components of streamflow. Since the residuals from each tree-ring regression with the SST anomalies will be highly correlated, a PCA is performed on the residual set, and components explaining 90% of the variance are retained$\left( {PC}_{{TR}_{\varepsilon}} \right)$. The retained PCs are regressed against the streamflow components due to local and regional transport and basin storage $\left( Q_{\varepsilon}^{*}\sim{PC}_{{TR}_{\varepsilon}} \right)$ to obtain the within basin streamflow component$\left( \hat{Q}_{\varepsilon}^{*} \right)$. The two streamflow component estimates $\left( \hat{Q}_{\varepsilon}^{*},\hat{Q}_{s}^{*} \right)$ are added together to obtain the AM transformed streamflow estimates$\left( \hat{Q}_{1}^{*} \right)$. This estimate is then transformed back to the original space with the inverse transformation of the HCDN basin, resulting in the AM streamflow reconstruction estimates $\left( \hat{Q}_{1} \right).$ Assuming ‘*p*’ number of principal components of $\left( {PC}_{{TR}_{\varepsilon}} \right)$ are retained, then the number of parameters under this regression is (*p+1*) including the intercept term. Thus, the total number of parameters, $m_{1},$ considered for building the AM is (*p+1*) +2 if ENSO/PDO alone influences and (*p+1*) +3 if both ENSO and PDO both influence the basin hydroclimate.

Adjusted R^2^, $\bar{R}^{2},$ are estimated based on the reconstructed streamflows from NM,$\hat{Q}_{0}$, and AM, $\hat{Q}_{1}$, by comparing with the observed streamflow ($Q)$. If the coefficient of determination (called as reduction error and coefficient of efficiency in dendrohydrology literature), R^2^, of the NM and AM are $R_{0}^{2}$ and $R_{1}^{2}$ respectively, then the adjusted R^2^ for the NM (${\bar{R}^{2}}_{0})$and AM (${\bar{R}^{2}}_{1})$ can be written as

$${\bar{R}^{2}}_{0}=1-\left[ \frac{(1-R_{0}^{2})(n-1)}{(n-m_{0}-1)} \right]$$

$${\bar{R}^{2}}_{1}=1-\left[ \frac{(1-R_{1}^{2})(n-1)}{(n-m_{1}-1)} \right]$$

where *n* denotes the number of years of reconstructed annual flows and *m_1_ = (p+1)* if ENSO/PDO influences and *m_1_= (p+2)* if both ENSO and PDO influence together.

The performances of AM and NM are also evaluated based on normalized root mean square error (N-RMSE – *0* denotes NM and *1* denotes AM) in predicting the annual flows using the following equation where $\bar{Q}$ denotes the mean annual flows for normalizing the root mean square error at a given site.

$$N\text{-}RMSE_{0/1}=\frac{\sqrt{n^{-1}\sum_{i=1}^{n} \left(Q_{i}-\hat{Q}_{i,0/1}\right)^{2}}}{\bar{Q}}$$

To calculate N-RMSE for above-normal flows, annual flows above 67^th^ percentile was considered with the normalization based on the mean of the above-normal flows.

Comparison between the AM and NM was considered only if the reconstructed flows by one of the models exhibit statistically significant skill with the observed flows. The statistically significance between the observed flows and the reconstructed flows was tested based on Pearson correlation, which is considered as significant if the estimated correlation is above 1.96/(n-3)^0.5^ where n denotes the number of years of observed annual flows. Out of 301 sites, 19 sites exhibited no skill in predicting the annual flows using both models (indicated in cyan in Figures 3 and 4).

**SI-4.0 Additional Discussion on Results**

As discussed in the main text, the hybrid AM approach shows higher reconstruction skill than the NM for the majority of HCDN basins in the CONUS. Of the 301 HCDN basins used in this study, the AM had a higher adjusted R^2^ value than the NM in 268 basins (89%). Similarly, the AM had a lower N-RMSE in 268 basins compared to 33 basins where the NM had a lower N-RMSE (Figure SI-3). Details on the 33 HCDN basins where the NM had a higher adjusted R^2^ value and lower N-RMSE are given in Table SI-1. Although the NM model performed better in these 33 basins, the improvement was only slight as only 17 basins had an adjusted R^2^ difference greater than 0.01 and only 9 basins had an adjusted R^2^ difference greater than 0.02. In comparison, the Adjusted R^2^ improvement of the AM from the NM in the other 268 sites averages 0.11, and the N-RMSE is larger than 0.1 in many sites (Figure SI-3). Evaluating the performance of AM in improving the prediction of above-normal flows based on N-RMSE shows that AM performs better 257 sites compared to 44 sites under the NM (Figure SI-4).

One similarity of many of the 33 basins where the NM outperforms the AM is smaller drainage areas and mean annual streamflow (Table SI-1). Figure SI-5 shows the reconstructed flows where NM performed better than AM for four stations. The improvement in predicting above-normal flows under these stations is not significant in comparison to the stations where AM performs better (Figure 6). Most of the basins where NM performs better have a drainage area smaller than 1000 square miles and have mean annual streamflow less than 1000 cfs (Figure SI-6). One of the limitations of tree-ring chronologies is the metabolic growth limit of a tree after saturation. Since SST anomalies do not have such a limitation, their addition can improve reconstruction skill. However, in smaller basins, it is natural to expect limited role of large-scale climate since the basin could be potentially influenced by local/regional hydroclimatology. Thus, using SST anomalies as a predictor may not result in any significant improvement in reconstructing the annual flows for smaller basins.

**SI-5.0 Lillefers Test**

The Lilliefors test [[6][7]] is a goodness-of-fit test to check whether the data is normally distributed or not, especially when the population mean and population standard deviation are not known ([3]). It offers an improvement over Kolmogorov-Smirnov test (K-S test), as for the K-S test population parameters must be specified. In Lilliefors test, we check the null hypothesis that the data follows a normal distribution against the alternative that the data does not come from a normal distribution. Steps involved in Lilliefors test are as follows:

1. At first, the z-score is calculated for each member in the sample.

$Z_{i}=\frac{X_{i}-\bar{X}}{s}$ , $i=1,2,\ldots,n$

where $Z_{i}$ are the z-score of individual data point $X_{i}$. $\bar{X}$ and *s* are the sample mean and sample standard deviation of the data.

1. The test statistic has the form

$T= {\text{sup} \atop x} \left| F\left( x \right)-S(x) \right|$

where *F(x)* and *S(x*) are the cumulative distribution functions of empirical distribution calculated based on sample data and the normal distribution with mean and standard deviation estimated from the sample data respectively.

1. For a desired significance level or confidence interval, the test statistic is compared with the critical value ([1]) for the test. In this case we used 95% confidence interval leaving 2.5% on the both tails of the distribution. It should be noted that Lilliefors test is a two-tailed test. The *p-*values are defined as the probability of observing a test statistic as extreme as or more extreme than the observed data under null hypothesis ([5]).

In Figure SI-7 the *p*-values for the Lilliefors test of annual streamflow are shown for the HCDN stations considered in this study. Smaller the *p-*value, less reliable is the null hypothesis. Depending on whether we do not reject or reject the null hypothesis, we comment on the normality of the sample data.

**SI-6.0 Box-Cox Transformation**

Box-Cox transformation ([2]) is a method of monotonic transformation using power function. Apart from the normality assumptions in regression methods, transformation of the annual streamflow series to normal flows help in handling skewness in the data. We apply Box-Cox transformation for the stations where annual streamflow series does not follow Normal distribution, as verified using Lilliefors test (refer Text S-5), to generate approximately normally distributed streamflow series.

$$T\left( Y \right)= \left\{ \begin{aligned} \frac{Y^{\lambda}-1}{\lambda}, \lambda\neq0 \\ \log\left( Y \right),\lambda=0 \end{aligned} \right.$$

Here *Y* is the observed annual streamflow series at HCDN sites and $\lambda$ is the parameter of transformation. Value of λ ranges from -5 to 5, each of which is tested so that transformed data is the best approximation of a normally distributed series. In Figure SI-8, the values of transformation parameter λ are plotted for each site. Shapes of the marker indicate which station’s annual streamflow is significantly correlated to which SST index. The colors indicate various categories of λ values.

**References**

1. Abdi, H. and Molin, P. (2007) Lilliefors/Van Soet's test of normality. In: Neil Salkind (Ed.) (2007), Encyclopedia of Measurement and Statistics
2. Box, G. E. P. and Cox, D. R. (1964). An analysis of transformations, *Journal of the Royal Statistical Society*, Series B, *26*, 211-252.
3. Conover, W. J. Practical Nonparametric Statistics. Hoboken, NJ: John Wiley & Sons, Inc., 1980.
4. Hamlet, A.F., Lettenmaier, D.P., 1999. Columbia River streamﬂow forecasting based on ENSO and PDO climate signals. J. Water Resour. Plann. Manage. 125, 333– 341.
5. Kutner, M., Nachtsheim, C., Neter, J., and Li, W. (2004). Applied Linear Statistical Models, McGraw-Hill/Irwin, Homewood, IL.
6. Lilliefors, H. W. "On the Kolmogorov-Smirnov test for normality with mean and variance unknown." *Journal of the American Statistical Association*. Vol. 62, 1967, pp. 399–402.
7. Lilliefors, H. W. "On the Kolmogorov-Smirnov test for the exponential distribution with mean unknown." Journal of the American Statistical Association. Vol. 64, 1969, pp. 387–389.
8. McCabe, G.J., Dettinger, M.D., 1999. Decadal variations in the strength of ENSO teleconnections with precipitation in the western United States. Int. J. Climatol. 19 (13), 1399–1410.
9. Patskoski, J., A. Sankarasubramanian and H. Wang, Reconstructed Streamflow using SST and Tree-ring Chronologies over the Southeastern United States, Journal of Hydrology, 527,761-775, 2015.
10. Slack, J. R., A. M. Lumb, and J. M. Landwehr. 1993a. Hydroclimatic data network (HCDN): A U.S. Geological Survey streamflow data set for the United States for the study of climate variation, 1874-1988. Water Resour. Invest. Rep., 93-4076.
11. Slack, J. R., A. Lumb, and J. M. Landwehr. 1993b. Hydro-Climatic Data Network (HCDN) Streamflow Data Set, 1874-1998. CD-ROM. U.S. Geological Survey, Reston, Virginia, U.S.A. Available from Oak Ridge National Laboratory Distributed Active Archive Center, Oak Ridge, Tennessee, U.S.A. [http://www.daac.ornl.gov].
12. Tootle, G.A., Piechota, T.C., 2006. Relationships between Paciﬁc and Atlantic Ocean Sea surface temperatures and U.S. streamﬂow variability. Water Resour. Res. 42, W07411.
